# A Case of COVID-19-Triggered Polymyositis Leading to Rhabdomyolysis

**DOI:** 10.7759/cureus.64267

**Published:** 2024-07-10

**Authors:** Sriya A Reddy, Natalie Rivera Vargas, Aarushi Varshney, Olga Karasik

**Affiliations:** 1 Internal Medicine, University of Central Florida (UCF) Hospital Corporation of America (HCA) Graduate Medical Education (GME) Consortium, Orlando, USA; 2 Internal Medicine, University of Central Florida College of Medicine, Orlando, USA

**Keywords:** idiopathic inflammatory myopathy, myalgia, rhabdomyolysis, polymyositis, covid19

## Abstract

SARS-COVID-19 is known to manifest with a wide variety of symptoms, most of which are respiratory. Myalgias are a common symptom of COVID-19, but cases of severe virus-induced inflammatory muscle injury leading to rhabdomyolysis and polymyositis have also been reported. Here, we present and discuss a case of a 56-year-old woman who presented with an initial presentation of COVID-19 infection with inflammatory polymyositis leading to rhabdomyolysis.

The patient was first treated for rhabdomyolysis with aggressive fluid resuscitation with intravenous normal saline without improvement in symptoms. She was then started on high-dose intravenous methylprednisolone for presumed immune-mediated polymyositis. An MRI of the bilateral lower extremities and a biopsy of the left thigh confirmed inflammatory myositis. After the initiation of steroids, liver function tests and creatinine kinase levels trended down, and symptoms improved. The patient was discharged with a prednisone taper and completely recovered at a follow-up six months later.

Post-COVID severe musculoskeletal involvement, including polymyositis or rhabdomyolysis, is rare, with only a few other cases published so far. Viral myositis, supported by myopathological evidence, should be considered carefully in patients with a recent COVID-19 infection after ruling out more common causes of myositis. Some proposed mechanisms include direct infection of the muscle or an environmental event triggering autoimmunity. Treatment generally involves corticosteroids that are gradually tapered.

## Introduction

COVID-19 has been known to manifest with a wide variety of symptoms, most of which are upper respiratory. Myalgias are a common symptom of COVID-19, and musculoskeletal manifestations of COVID-19 can range from muscle pain with mild or no weakness and mild elevation of creatinine kinase (CK) to severe rhabdomyolysis [1]. In rare cases, rhabdomyolysis can be the initial presentation. COVID-19 has been found to induce an exaggerated host immune response, leading to the marked production of autoantibodies; however, the precise mechanism by which virus-induced inflammatory muscle injury occurs is still unclear. Some proposed mechanisms are direct infection of the muscle or COVID-19 infection-causing environmental events triggering autoimmunity [2].

Rhabdomyolysis is a clinical syndrome caused by myocyte necrosis and the release of intracellular components like aldolase, myoglobin, CK, lactate dehydrogenase, and aspartate transaminase into the circulation, leading to acute kidney injury or fatal conditions such as arrhythmia or acute renal failure. In the setting of COVID-19 infection, rhabdomyolysis has been shown to increase the rates of mechanical ventilation, renal replacement therapy, and mortality [3]. This makes it important for physicians to have a high index of suspicion for rhabdomyolysis in patients with COVID-19 infection and persistent myalgias.

Here, we present a 56-year-old woman who presented with an initial diagnosis of COVID-19 and inflammatory polymyositis leading to rhabdomyolysis.

## Case presentation

A 56-year-old woman with a past medical history of alopecia, hepatitis A infection, and uterine fibroids (status post myomectomy) presented to the hospital with an upper respiratory tract infection. Symptoms were described as dyspnea and nasal congestion, associated with fever, generalized malaise, rigors, bilateral severe thigh muscle contractions, and dark-colored urine for three days. She denied recent sick contacts, seizure-like activity, trauma, strenuous exercise, or being immobilized for a prolonged period. She denied statin use; her only home medication was a multivitamin. In the emergency room, she was hypertensive at 152/106, and her remaining vital signs were within normal limits.

On physical examination, the patient had a regular rate and rhythm and normal heart sounds. Her lungs were clear for auscultation, and she was aerating well. Motor strength was 5/5 in bilateral upper and lower extremities, and there was generalized muscle tenderness. During the evaluation of the lumbar spine range of motion, limitations of lumbar flexion were noted; however, the lumbar extension was within normal limits. She was able to stand from a seated position and raise her arms above her head without difficulty. No rashes or lesions were noted on the skin.

The patient was found to be positive for COVID-19 infection on admission and Influenza A and B negative. Pertinent initial labs and trends over one week are illustrated in Table 1. Urinalysis showed amber-colored urine with large amounts of blood and no RBCs, consistent with rhabdomyolysis.

**Table 1:** Trend of pertinent laboratory values over one week

Laboratory Test	Reference Range	Day 1	Day 3	Day 5	Day 7
Creatinine	0.55-1.3 mg/dl	0.94	0.68	0.80	0.89
Potassium	3.7-5.1 mmol/L	4.0	4.1	4.2	4.1
Calcium	8.4-10.1 mg/dl	9.7	8.4	9.8	8.7
Bicarbonate	21-32 mmol/L	29	26	26	26
Creatinine Kinase	35-232 unit/L	>50000	>50000	>50000	2327
Aspartate Transaminase	10-27 unit/L	1128	1753	1520	589
Alanine Transaminase	12-78 unit/L	292	452	825	715
Alkaline Phosphatase	45-117 unit/L	74	55	72	61
Lactate dehydrogenase	100-190 unit/L	5279	2879	-	-
Erythrocyte Sedimentation Rate	0-30 mm/hr	25	-	-	-
C- Reactive Protein	0.290-19.0 mg/dl	0.560	-	-	-
Thyroid Stimulating Hormone	0.36-3.74 uIU/mL	0.68	-	-	-

During the initial inpatient stay, aggressive fluid resuscitation was initiated with a goal urine output of 1-3mL/kg/hr and intravenous (IV) normal saline of 0.9%. Renal and liver function, along with creatinine kinase (CK) labs, were trending. Despite aggressive IV fluid administration of over 2 liters a day for five days, the CK level stayed over 50,000 U/L, and transaminases continued trending up while serum creatinine and potassium stayed within normal limits (Table 1). She continued to endorse myalgias, primarily in the bilateral thigh and lumbar regions. 

**Figure 1:**
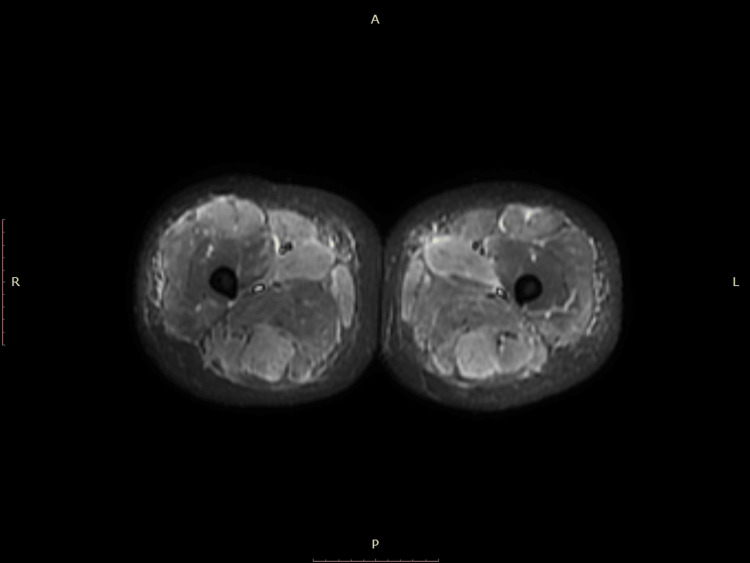
MRI axial STIR image of bilateral lower extremities showing muscle inflammation

As the patient was not responding to standard treatment of rhabdomyolysis, further workup with MRI of bilateral lower extremities with and without contrast illustrated mild myositis of bilateral adductor longus muscle, biceps, semitendinosus, and semimembranosus muscles (Figure 1). Due to the lack of expected response to aggressive IV fluids, we treated the patient on day five of admission with a high dose of methylprednisolone, 1000 mg IV per day for five days, to control the inflammation caused by the myositis, which, in turn, was causing rhabdomyolysis. After the initiation of steroids on day five, transaminases and CK levels started trending down, and symptoms began to improve (Table 1). 

A left thigh biopsy was obtained, which illustrated myopathic features with evidence of significant myofiber degeneration and regeneration along with mild T lymphocyte infiltration. Scattered “ring fibers” were noted. An autoimmune myositis panel was negative other than a positive ANA (anti-nuclear antibody) without a titre and an equivocal anti-ENA (extractable nuclear antibody) screen (Table 2). 

**Table 2:** ANA immunofixation panel Ab: Antibody; ANA: Antinuclear antibody; ENA: Extractable nuclear antigen; dsDNA: Double stranded DNA; IgG: Immunoglobulin G

Laboratory Test	Result
ANA pattern	Positive
Anti-ENA screen	1.1 (0-0.9 AI)
Jo-1 Ab	<0.2 (0-0.9 AI)
Scl-70 Ab (Topoisomerase1)​​​	<0.2 (0-0.9 AI)
dsDNA Ab	<1 (0-4.9 AI)
Anti-centromere Ab	<0.2 (0-0.9 AI)
Chromatin Ab	<0.2 (0-0.9 AI)
Acetylcholine receptor binding Ab	<0.03 (0-0.24 AI)
Rheumatoid Factor	<10 (IU/mL)
IgG4	10 (2-96 mg/dl)

As myositis secondary to the COVID-19 infection was suspected, the patient was discharged home on a prednisone taper dose for another five days along with pantoprazole. Six months later, the patient was evaluated by her primary care doctor for a regular follow-up. The review of symptoms was negative except for bilateral intermittent lower extremity muscle contraction. The respiratory, cardiovascular, and musculoskeletal physical exams were unremarkable. Labs, including kidney and liver function, were within normal limits.

## Discussion

The manifestations of COVID-19-induced myositis can exhibit a spectrum of presentations, spanning from muscle weakness to the typical features of dermatomyositis, including classic rashes or mild back pain with observable muscle abnormalities on MRI [1].

A systematic review of case reports on rhabdomyolysis and myositis estimated the incidence of rhabdomyolysis in hospitalized COVID-19 patients to be 0.2 to 2.2% [1]. Another study reported a mortality rate of 45% in patients who presented with rhabdomyolysis during short-term follow-up. Asymptomatic CK elevation is estimated to be present in as many as 16-33% of patients with musculoskeletal symptoms [2]. Although rhabdomyolysis is an infrequent complication of COVID-19, considering that CK elevation has been associated with an increase in mortality and a poor prognosis, CK levels should be assessed in patients experiencing musculoskeletal symptoms.

In this case report, we have referred to muscle injury as myositis/rhabdomyolysis yet there is discord in the nomenclature in the literature: post-viral myositis, viral-induced muscle injury, myopathy, myositis, and rhabdomyolysis are all commonly used terms [4]. Confusion over terminology may result in a lack of reporting and diagnosis of muscle injury events. Our patient satisfied four of the required criteria for the diagnosis of polymyositis (elevated creatinine kinase, muscle pain upon grasping and spontaneous muscle pain, nondestructive arthritis and arthralgias, and muscle biopsy findings compatible with inflammatory myositis), leading to the definitive diagnosis of polymyositis as per the Myositis Association [5]. 

Several potential mechanisms have been suggested to explain the muscle inflammation triggered by COVID-19, including direct virus entry into muscle cells via ACE-2 receptors, virus-triggered autoinflammation or exaggerated host immune response with autoantibody production, induction of a hyperinflammatory state, and even molecular mimicry [2]. Additional research is necessary to comprehensively grasp the pathogenesis. 

A review of 26 other cases in the literature shows varied demographics, comorbidities, treatment plans, and responses to treatments and highlights COVID-induced polymyositis as an important cause of rhabdomyolysis in patients who are COVID-positive [6-9]. In terms of treatment, most cases in the literature were treated with corticosteroids that are gradually tapered. This patient had a good response to corticosteroids. Immune suppressive therapy has been effective if there is an unsatisfactory response to steroids [3]. Intravenous immunoglobulins (IVIG) can be considered for chronic and refractory cases [10].

Although in this case the diagnosis of inflammatory myositis was made certain with MRI and muscle biopsy, limitations include a lack of weakness and the lack of a consistent definition of COVID-19-induced muscle injury in the literature. Rhabdomyolysis should be suspected when the symptom triad of muscle pain, weakness, and dark-colored urine is present, but few patients have all three symptoms [11].

The presentation of rhabdomyolysis secondary to inflammatory myositis is important to distinguish rhabdomyolysis from other causes (overuse/crush injury), as the latter responds quickly to aggressive IV fluids with a decline in CK levels in the 3-5 days following cessation of muscle injury [12]. On the other hand, rhabdomyolysis secondary to myositis requires treatment directed at stopping muscle injury with steroids and should be suspected when the patient clinically and objectively does not respond to aggressive IV fluid therapy and a cause of myositis (virus-mediated injury in this case) is plausible.

## Conclusions

Post-COVID-19 severe musculoskeletal involvement, including polymyositis or rhabdomyolysis, is rare, with only a few other cases published so far. The mechanism by which the musculoskeletal system is affected by COVID-19 may include an exaggerated host immune response but remains unclear and requires further studies. CK elevation is associated with an increase in mortality and poor prognosis and should be routinely checked in COVID-19-positive patients with severe musculoskeletal symptoms. Testing for myopathy with antibody testing, MRI, and muscle biopsy should be considered when there is concern for specific myositis.
